# Complete genome sequence of Zoysia mosaic virus from Japan

**DOI:** 10.1128/mra.01121-25

**Published:** 2026-03-23

**Authors:** Yutaro Neriya, Kimathi Harun Ringeera, Hisashi Nishigawa

**Affiliations:** 1 School of Agriculture, Utsunomiya University 13144, , Utsunomiya, Tochigi, Japan; 2 Department of Biological Production Science, United Graduate School of Agricultural Science, Tokyo University of Agriculture and Technology 13125, Fuchu, Tokyo, Japan; Katholieke Universiteit Leuven, Leuven, Belgium

**Keywords:** *Potyviridae*, *Poacevirus*, Zoysia

## Abstract

We report a complete genome sequence of Zoysia mosaic virus (ZoMV) from Japanese lawngrass. This virus shares around 80% nucleotide identity with the previously reported ZoMV-ZM864 isolate from Manila grass.

## ANNOUNCEMENT

Zoysia mosaic virus (ZoMV) is a pathogen responsible for mosaic symptoms in Zoysia grasses. It was first reported in Japan more than 50 years ago (1). Initially, based on virion morphology, it was suspected as a member of the genus *Potyvirus*. However, in 2023, sequencing of the ZoMV-ZM864 isolate (DDBJ/ENA/GenBank accession number OP425115) obtained from Manila grass (*Zoysia matrella*) exported from Japan to the United States suggested that the virus belongs to the family *Potyviridae* and genus *Poacevirus* (2). In this study, we determined the complete genome sequence of a ZoMV (ZoMV-TCG) infecting Japanese lawngrass (*Z. japonicum*) exhibiting mosaic symptoms collected in Nasu-Shiobara City, Tochigi Prefecture in 2022.

Double-stranded RNA (dsRNA) was extracted using a micro-spin column containing cellulose D powder (Advantec, Japan) (3) from symptomatic Japanese lawngrass. A template-switching RT enzyme mix (NEB) was used to synthesize complementary DNA (cDNA) with this dsRNA and TSO (5′- AAGCAGTGGTATCAACGCAGAGTACATrGrGrG-3′, rG: guanine ribonucleotide, underline: common sequence according to procedure protocol: https://www.neb.com/ja-jp/protocols/5-race-protocol-using-the-template-switching-rt-enzyme-mix) as template and primer TSO-F1-N6 (5′-AAGCAGTGGTATCAACGCAGAGTTACANNNNNN-3′) as a primer. Since the synthesized cDNA contains a common sequence at its 5′ end and a sequence complementary to the common sequence at its 3′ end, KOD One (TOYOBO, Japan) PCR was performed using AUAP-TSO-F1-in5 primer (5′-GGCCACGCGTCGACTAGTACACAGTGAAGCAGTGGTATCAACGCAGAGT-3′) with the common sequence at its 3′ end. The amplified product was purified using a NucleoSpin Gel and PCR Clean-Up Kit (Takara Bio, Japan), ligated with ligation sequencing DNA V14 (SQK-LSK114, Oxford Nanopore Technologies (ONT), UK), and subjected to whole-genome sequencing with a MinION Mk1B and Flongle Flow Cell (R10.4.1) (ONT), generating 465,063 reads (average read length: 1,241.3). Nanopore sequencing reads containing both expected 5′ and 3′ terminal adapter sequences were extracted using Cutadapt with an allowed error rate of 10%, and reads lacking either adapter were discarded (4). Adapter-filtered reads were further quality- and length-filtered using NanoFilt (minimum Q10 and 200 bp) (5). Filtered reads were directly mapped to ZoMV-ZM864 using minimap2 (6), followed by one round of consensus polishing with Racon (7). Final error correction was performed using Medaka (8). Default parameters were used in these analyses unless otherwise specified.

**Fig 1:**
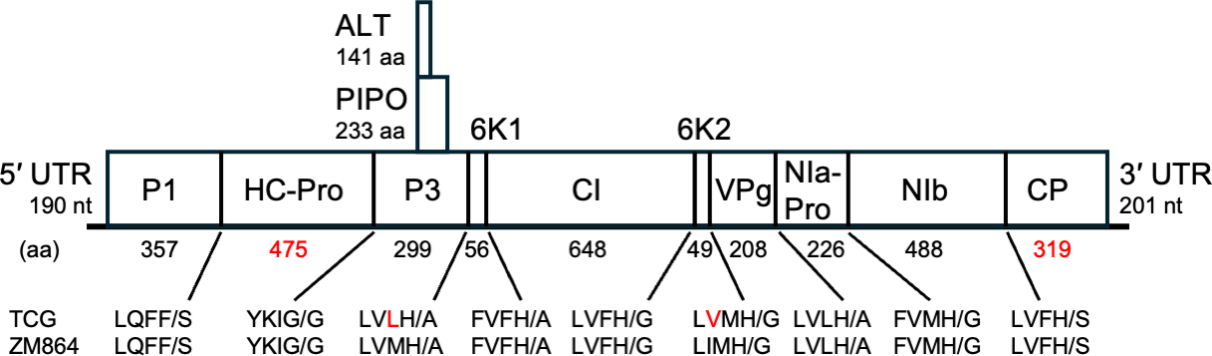
Schematic overview of Zoysia mosaic virus-TCG showing the polyprotein and the mature protein products. The putative cleavage sites in the ZoMV polyproteins are indicated below the genome. Red letters represent differences between ZoMV-TCG and ZoMV ZM864 isolates.

The complete genome of ZoMV-TCG was determined to be 9,766 nucleotides (nt) in length, excluding the 3′-terminal poly(A) tail, with a guanine or cytosine (GC) content of 40.3%. The average genome coverage of sequences mapped to the ZoMV sequence was 6,666.42-fold. Similar to ZoMV-ZM864, a single large open reading frame (ORF) spanning nt 191–9,565 encoding a polyprotein of 353.9 kDa was predicted using the GENETYX-MAC v22.0.5 (Fig. 1). This polyprotein contained nine potential potyvirus cleavage sites, consistent with the previously reported ZoMV-ZM864 isolate. Additionally, small ORFs, called P3N-PIPO (9) and P3N-ALT (10), were also predicted to encode products generated by transcriptional slippage at the GA_8_ motif (nt 3,090–3,098). Sequence alignments with ZoMV-ZM864 were performed using the MUSCLE algorithm (11) in GENETYX-MAC under default parameters. The ZoMV-TCG ORF of polyprotein shared 79.6% nucleotide identity and 90.6% amino acid identity. According to the classification criteria of the *Potyviridae* family (12), ZoMV-TCG was confirmed to be an isolate of ZoMV.

## Data Availability

The genome sequences of ZoMV-TCG were deposited in the DDBJ/ENA/GenBank database under accession number LC895292. The MinION data set was deposited in the NCBI Sequence Read Archive (SRA) under BioProject accession number PRJDB37752 and SRA run accession number DRR786955.

## References

[1] Toriyama S, Yora K, Asuyama H. 1968. Three types of stranded viruses detected in Gramineae plants. Ann Phytopath Soc Japan 34:199. doi:10.3186/jjphytopath.34.163

[2] Adhikari BN, Zhou J, Hu X, Turner RS, McFarland C, Foster JA. 2023. Complete genome sequence of zoysia mosaic virus, a novel member of the genus Poacevirus. Arch Virol 168:136. doi:10.1007/s00705-023-05763-0 37043050

[3] Okada R, Kiyota E, Moriyama H, Fukuhara T, Natsuaki T. 2015. A simple and rapid method to purify viral dsRNA from plant and fungal tissue. J Gen Plant Pathol 81:103–107. doi:10.1007/s10327-014-0575-6

[4] Martin M. 2011. Cutadapt removes adapter sequences from high-throughput sequencing reads. EMBnet J 17:10. doi:10.14806/ej.17.1.200

[5] De Coster W, D’Hert S, Schultz DT, Cruts M, Van Broeckhoven C. 2018. NanoPack: visualizing and processing long-read sequencing data. Bioinformatics 34:2666–2669. doi:10.1093/bioinformatics/bty149 29547981 PMC6061794

[6] Li H. 2018. Minimap2: pairwise alignment for nucleotide sequences. Bioinformatics 34:3094–3100. doi:10.1093/bioinformatics/bty191 29750242 PMC6137996

[7] Vaser R, Sović I, Nagarajan N, Šikić M. 2017. Fast and accurate de novo genome assembly from long uncorrected reads. Genome Res 27:737–746. doi:10.1101/gr.214270.116 28100585 PMC5411768

[8] Oxford Nanopore Technologies Ltd. 2025. Medaka: sequence correction provided by ONT. Available from: https://github.com/nanoporetech/medaka

[9] Chung BYW, Miller WA, Atkins JF, Firth AE. 2008. An overlapping essential gene in the Potyviridae. Proc Natl Acad Sci U S A 105:5897–5902. doi:10.1073/pnas.0800468105 18408156 PMC2311343

[10] Hagiwara-Komoda Y, Choi SH, Sato M, Atsumi G, Abe J, Fukuda J, Honjo MN, Nagano AJ, Komoda K, Nakahara KS, Uyeda I, Naito S. 2016. Truncated yet functional viral protein produced via RNA polymerase slippage implies underestimated coding capacity of RNA viruses. Sci Rep 6:21411. doi:10.1038/srep21411 26898356 PMC4761962

[11] Edgar RC. 2004. MUSCLE: multiple sequence alignment with high accuracy and high throughput. Nucleic Acids Res 32:1792–1797. doi:10.1093/nar/gkh340 15034147 PMC390337

[12] Inoue-Nagata AK, Jordan R, Kreuze J, Li F, López-Moya JJ, Mäkinen K, Ohshima K, Wylie SJ, ICTV Report Consortium. 2022. ICTV virus taxonomy profile: Potyviridae 2022. J Gen Virol 103:001738. doi:10.1099/jgv.0.001738 35506996 PMC12642016

